# Supplementary material for: Analysis of common differential gene expression in synovial cells of osteoarthritis and rheumatoid arthritis
Source: PLoS One. 2024 May 21;19(5):e0303506. doi: 10.1371/journal.pone.0303506 (PMC11108184; doi:10.1371/journal.pone.0303506)
Supplement: Attachment — Submitted filename: Response to Reviewers.docx [file pone.0303506.s002.docx]

Dear Reviewers,

We would like to express our sincere gratitude for the valuable feedback provided on our manuscript. We have carefully considered each of the major comments and have made the necessary revisions to improve the clarity and quality of our work. Below is our point-by-point response to the major comments:

1.We have expanded the description of the datasets in the manuscript. We have now included details such as the type of dataset (Microarray/transcriptome), the number of samples, sample size (control and Disease), geographical origin, age group/gender information available, and the keywords used to extract the datasets.

2.On page 11, we have clarified the description of the algorithms, tools, and packages used for the identification of differentially expressed genes (DEGs). We have specified the software and versions used for the analysis.

3.We apologize for the confusion caused by the term "transcriptional data" in line 12 to 14 of page 11. We have now corrected it to "gene expression data" to avoid any ambiguity.

4.We have revised lines 21–23 of page 11 to ensure that the presentation of results is distinct from the methodology and does not cause any confusion.

5.We have updated the description of biological pathways on page 12, line 4, to clearly state that gene ontology (GO) identifies biological processes, cellular components, and molecular functions.

6.On page 12, line 11, we have now described the comprehensive process of hub gene identification, which includes the merging of several topological parameters such as degree, closeness, betweenness, maximum clique centrality (MCC), and others. We have ensured that the criteria for identifying hub genes are justified and not based on a single network characteristic.

7.We have revised the introduction to remove unnecessary details and to maintain a clear focus on the context of our current study.

8.On page 12, line 21, we have rephrased the sentence to accurately describe the validation analysis as an integral part of our study.

9.We have plotted and provided a density plot to confirm that all data have been uniformly standardized.

10.Thank you for your careful review of our research. Regarding the issue you mentioned about the number of 15 common differentially expressed genes (DEGs), this figure is based on our bioinformatics analysis results. In our analysis of gene expression data, we adopted strict statistical criteria and bioinformatics tools to identify genes that are either up-regulated or down-regulated in synovial cells from patients with osteoarthritis (OA) and rheumatoid arthritis (RA). These criteria include the magnitude of gene expression changes, statistical significance levels, and the relevance of biological functions.During the analysis, we found that 15 genes showed upregulated expression in both diseases, while another 15 genes exhibited downregulated expression. This number is not preset but a natural outcome of the data analysis. The discovery of these common DEGs provides us with important clues to investigate the shared pathological mechanisms of these two diseases and may offer new targets for future therapeutic strategies.We understand that this number may seem like a coincidence, but it indeed reflects the authentic results of our analysis. We believe that the discovery of these common DEGs is significant for understanding the pathophysiology of OA and RA, and we will continue to delve deeper into the functions of these genes and their roles in the diseases.

11.We have improved the clarity of the Venn diagram to ensure that it effectively conveys the necessary information.

12.On page 16, we have corrected the term "Biological Pathway (BP)" to "Biological Process (BP)" to avoid confusion with KEGG pathway analysis.

13.We have enhanced the visual clarity of the network displayed on page 18 by optimizing the layout and mentioning the number of nodes and edges in the text.

14.We have shortened the figure legends on pages 17, 21, 22, and 24 for brevity and clarity.

15.We have restructured the discussion section to present our findings more effectively and have cited additional research to support our conclusions.

16.In the conclusion, we have revised the statement to improve its readability and grammatical correctness. We have also provided a justification for our findings, emphasizing their significance in understanding the molecular mechanisms shared by osteoarthritis (OA) and rheumatoid arthritis (RA), and their implications for therapeutic strategies.

Minor Comment:

1.Regarding the spelling error - We have corrected "Founding" to "Funding" on page 26, line 22. Thank you for pointing out this mistake.

2.Regarding the clarity of subfigure labeling - We have redesigned all relevant subfigures to ensure that the labels are placed within the graphics themselves, enhancing visual appeal and clarity. These changes have been updated on pages 9, 10, 11, 12, 15, and 16.

3.Regarding clinical studies/case reports - We have added more clinical studies and case reports related to the association between osteoarthritis and rheumatoid arthritis to support our results and discussion. These newly added references can be found in the reference list.

4.Regarding the restatement of the research objective - We acknowledge that the original statement of the research objective may have caused some confusion. We have rephrased the research purpose to more accurately reflect our findings, which identified common differentially expressed genes between osteoarthritis and rheumatoid arthritis, rather than for differential diagnosis between the two. This modification has been updated on lines 4 to 7 of page 1.

Once again, we appreciate the time and effort spent by the reviewers in evaluating our manuscript. We believe that the revisions addressed in this response have significantly improved our work, and we hope that it is now suitable for publication.

Sincerely,

Changsheng-liao
